# SG-APSIC1137: 12-hydroxystearic acid upregulates skin antimicrobial peptides in skin models and provides long-lasting protection from bacterial challenge from a handwash formulation

**DOI:** 10.1017/ash.2023.50

**Published:** 2023-03-16

**Authors:** Morris Waskar, Xuelan Gu, Tingyan Mi, Meenakshi Swaminathan, Carol Vincent, Rimpa Ghosh, Chandraprabha Doraiswamy, Amitabha Majumdar

**Affiliations:** Unilever, India; Unilever, Shanghai, China; Unilever, Shanghai, China; Unilever, Mumbai, India; Unilever, Trumbull, Connecticut, United States; Unilever, Mumbai, India; Unilever, Bangalore, India; Unilever, Bangalore, India

## Abstract

**Objectives:** We evaluated the role of 12-hydroxystearic acid (12HSA) in upregulating skin antimicrobial peptides (AMPs) in in vitro and ex vivo assays, and 12HSA provides long-lasting germ protection in vivo through a handwash formulation. **Methods:** In vitro assays were performed by treating skin cells, maintained in cell-culture media, with 12HSA. After treatment, AMP gene-expression was measured in cells by RT-qPCR, and secreted AMPs in spent cell culture media were analyzed by ELISA. Skin explants were treated with 12HSA, and 3D-living skin equivalent (LSE) models were treated with 12HSA-containing handwash formulations. AMP levels were measured by immunohistochemical staining or RT-qPCR after treatment. In clinical studies, volunteer forearms were washed multiple times with 12HSA-containing handwash in an ethics-approved study in which participants provided informed consent. The washed forearms were challenged with *E. coli* at different time points after washing. The 12HSA deposition from the formulation was measured using tape strips. **Results:** Skin cells treated with 12HSA showed increased expression of several AMP genes in vitro, and higher psoriasin AMP secretion was measured in cell-culture media. An enhanced level of LL37 AMP was obtained from the skin epidermis of 12HSA-treated explant skin. AMP genes were also upregulated in the 3D-LSE model treated with a 12HSA-containing handwash formulation. A measurable level of 12HSA was deposited from handwash formulation in the in vivo clinical sample. *E. coli* recovery from challenged skin was significantly lower at 6 and 10 hours after washing compared to unwashed skin. **Conclusions:** These data demonstrate that 12HSA boosts skin-AMPs and that a handwash containing 12HSA provides long-lasting germ protection under in vivo test conditions by potentially enhancing skin’s natural immunity. With an emerging understanding of skin’s innate immunity and AMPs, designing cleansing products that strengthen these natural defenses will offer novel approaches to extend hygiene benefits beyond immediate in-wash protection.

